# Secondary prevention of cardiovascular disease in Brazil: lessons from the Brazilian Longitudinal Study of Adult Health (ELSA-Brasil)

**DOI:** 10.1590/1516-3180.2019.1376091219

**Published:** 2020-03-06

**Authors:** Isabela Martins Benseñor, Paulo Andrade Lotufo

**Affiliations:** I MD, PhD. Full Professor, Department of Internal Medicine, Faculdade de Medicina FMUSP, Universidade de Sao Paulo, São Paulo (SP), Brazil.; II MD, DrPH. Full Professor, Department of Internal Medicine, Faculdade de Medicina FMUSP, Universidade de Sao Paulo, São Paulo (SP), Brazil.

In the 21^st^ century, cardiovascular disease is the greatest cause of mortality worldwide, considering coronary heart disease and stroke together. It is also the leading cause of years of life lost, both worldwide and in Brazil.

Secondary prevention of cardiovascular disease encompasses all strategies such as changes in lifestyle, use of medications to treat cardiovascular disease and associated risk factors and rehabilitation after an event, among people who have previously suffered myocardial infarction or stroke. The objective is simple and clear: to prevent new cardiovascular events in a high-risk group, as proposed by Geoffrey Rose in the 1970s.^1^

Secondary prevention of coronary heart disease and stroke, especially using pharmacological therapy, has been effective in reducing the number of new fatal or nonfatal events worldwide. However, information about secondary prevention of cardiovascular diseases in Brazil is scarce. Previous studies in Brazil have identified the most formidable barriers preventing access to cardiac rehabilitation, which include transportation difficulties, low income, lack of insurance coverage and low educational level.^2^^,^^3^^,^^4^ Regarding stroke, a previous study showed that less than 50% of patients with stroke who are attended in public hospitals and healthcare centers have any medical follow-up after their hospitalization at the time of the event.^5^

Recent data from the Brazilian Longitudinal Study of Adult Health (ELSA-Brasil) have presented similar results.^6^^,^^7^ ELSA-Brasil is a prospective cohort study of 15,105 civil servants in six cities in Brazil (Belo Horizonte, Porto Alegre, Rio de Janeiro, Salvador, São Paulo and Vitória). In this sample, 197 individuals (1.3%) reported at the baseline that they had previously had a stroke. Among them, 20% had then not used any medication for secondary prevention of stroke. Fifteen years after the event, only 39 participants (19.8%) were still using some type of medication for stroke prevention. The same was valid for secondary prevention of coronary heart diseases. Out of the 405 participants who reported at the baseline that they had previously had myocardial infarction, only 35% reported that they had then used drugs for secondary prevention of coronary heart disease. Drug use was more common among high-income participants than among low-income participants. Fifteen years after the stroke event, only 46 (11.6%) of the participants with a previous myocardial infarction were still using some type of medication for secondary prevention of coronary heart disease. Moreover, both studies highlighted a critical point: secondary prevention was less used among women than among men.^6^^,^^7^

This lack of secondary cardiovascular prevention is not a scientific secret, and also it is not an exclusively Brazilian problem. Some other studies have shown lower frequency of use of medication for secondary prevention of cardiovascular disease among women than among men.^8^^,^^9^ Previous data from ELSA-Brasil showed that women were more conscious about their health status than men were, but also showed that they were receiving less prescription of medication for secondary prevention of coronary heart disease from healthcare professionals.^10^^,^^11^

The reasons that can explain these findings include higher frequency of atypical symptoms among women than among men, along with some underestimation of the severity of the disease in women. The pattern of statin use among the women in the ELSA-Brasil sample is similar to the pattern reported in the Reasons for Geographic and Racial Differences in Stroke (REGARDS) study. In that study, statin use was highest among white men, followed by black men, white women and black women.^8^ Data from another study conducted in primary care settings also showed that more than 50% of primary care physicians did not use risk stratification tools in clinical practice, and that this may have led to lower frequency of statin use, especially among women.^9^

ELSA-Brasil also presented important data correlating the use of medication for secondary prevention with socioeconomic status. In both studies,^6^^,^^7^ the use of secondary prevention was higher among participants with high socioeconomic status than among participants with low socioeconomic status. One crucial point needs to be noted here: the sample for ELSA-Brasil comprised civil servants with high educational attainment and monthly family income, compared with the general population in Brazil. Therefore, it can be suggested that the use of secondary prevention in the general population of Brazil, which has lower educational attainment and monthly family income than the ELSA-Brasil sample, is probably worse.

What is done for patients with myocardial infarction or stroke after they have been discharged from their hospitalization? It seems that not much is done for them, or at least much less than necessary. The solution lies in ensuring that every patient who has had a cardiovascular event is provided with an adequate level of follow-up with further access to secondary prevention. Achieving this is not easy, but it is the only thing to do right now.

## References

[1] Rose G (1981). Strategy of prevention: lessons from cardiovascular disease. Br Med J (Clin Res Ed).

[2] Lotufo PA (2017). Cardiovascular secondary prevention in primary care setting: an immediate necessity in Brazil and worldwide. Sao Paulo Med J.

[3] Ghisi GL, dos Santos RZ, Aranha EE (2013). Perceptions of barriers to cardiac rehabilitation use in Brazil. Vasc Health Risk Manag.

[4] Borghi-Silva A, Mendes RG, Trimer R, Cipriano G (2014). Current trends in reducing cardiovascular disease risk factors from around the world: focus on cardiac rehabilitation in Brazil. Prog Cardiovasc Dis.

[5] Cabral NL, Franco S, Longo A (2012). The Brazilian Family Health Program and secondary stroke and myocardial infarction prevention: a 6-year cohort study. Am J Public Health.

[6] Abreu FG, Goulart AC, Birck MG, Benseñor IM (2018). Stroke at baseline of the Brazilian Longitudinal Study of Adult Health (ELSA-Brasil): a cross-sectional analysis. Sao Paulo Med J.

[7] Birck MG, Goulart AC, Lotufo PA, Benseñor IM (2019). Secondary prevention of coronary heart disease: a cross-sectional analysis on the Brazilian Longitudinal Study of Adult Health (ELSA-Brasil). Sao Paulo Med J.

[8] Gamboa CM, Colantonio LD, Brown TM, Carson AP, Safford MM (2017). Race-Sex Differences in Statin Use and Low-Density Lipoprotein Cholesterol Control Among People with Diabetes Mellitus in the Reasons for Geographic and Racial Differences in Stroke Study. J Am Heart Assoc.

[9] Mosca L, Linfante AH, Benjamin EJ (2005). National study of physician awareness and adherence to cardiovascular disease prevention guidelines. Circulation.

[10] Chor D, Pinho Ribeiro AL, Sá Carvalho M (2015). Prevalence, Awareness, Treatment and Influence of Socioeconomic Variables on Control of High Blood Pressure: Results of the ELSA-Brasil Study. PLoS One.

[11] Lotufo PA, Santos RD, Figueiredo RM (2016). Prevalence, awareness, treatment, and control of high low-density lipoprotein cholesterol in Brazil: Baseline of the Brazilian Longitudinal Study of Adult Health (ELSA-Brasil). J Clin Lipidol.

